# ‘…completely exhausted and weak, really drained.’: a multinational qualitative interview study of healthcare provider, patient, and caregiver experiences of C3 glomerulopathy and primary (idiopathic) immune complex membranoproliferative glomerulonephritis diagnosis, burden and management

**DOI:** 10.3389/fneph.2026.1770706

**Published:** 2026-05-22

**Authors:** Sayna Norouzi, Mingyi Huang, Carly Rich, Katie Gordon, Lucia Quintana-Gallardo, Ryan Naylor, Mona Amet, Laura Mirams, Elizabeth Holdsworth, Fernando Caravaca-Fontán

**Affiliations:** 1 Division of Nephrology, Loma Linda University Medical Center, Loma Linda, CA, United States; 2 Apellis Pharmaceuticals, Inc., Waltham, MA, United States; 3 Swedish Orphan Biovitrum AB, Stockholm, Sweden; 4 Adelphi Real World, Bollington, United Kingdom; 5 Department of Nephrology, Instituto de Investigación Hospital 12 de Octubre (imas12), Madrid, Spain

**Keywords:** C3G, caregiver burden, glomerulonephritis, healthcare professionals, patient burden, primary IC-MPGN

## Abstract

**Introduction:**

C3 glomerulopathy (C3G) and primary (idiopathic) immune complex membranoproliferative glomerulonephritis (IC-MPGN) are rare progressive kidney diseases. Due to the rare nature of C3G/primary IC-MPGN, there is a paucity of real-world data on the diagnosis, management, and impact of the diseases on patients and caregivers. Our study used the direct experiences of healthcare professionals (HCPs), patients, and caregivers, to provide insight into the diagnosis/management of C3G/primary IC-MPGN and its burden on patients and caregivers.

**Methods:**

We conducted a multi-country, cross-sectional, semi-structured, qualitative interview study with HCPs, patients, and caregivers in France, Germany, Italy, Spain, the United Kingdom and the United States between July 2024−January 2025. Nephrologists or internal medicine specialists (France and Spain only) were eligible to participate if they managed at least one patient with a biopsy-confirmed C3G/primary IC-MPGN diagnosis. Patients and informal caregivers were recruited via HCPs, patient advocacy groups, online panels or social media and had to be aged ≥16 years and ≥18 years respectively. Thematic analysis was conducted to identify key response themes.

**Results:**

A total of 21 HCPs, 13 patients, and 8 caregivers were interviewed. HCPs and patients highlighted that milder symptoms often led to diagnostic delays with HCPs highlighting lack of disease recognition, difficulties accessing specialist care, and patient reluctance to receive a biopsy as barriers to timely diagnosis. Mental and physical fatigue/exhaustion were the most burdensome symptoms according to patients and caregivers, impacting all aspects of the patient’s life. Patients stated that achieving disease stability was a key goal of treatment, however HCPs, patients, and caregivers all stated that treatment options at survey (prior to approval of iptacopan and pegcetacoplan) were limited, with dialysis being particularly burdensome to patients. HCPs were hopeful about future treatment options. Caregivers reported that they provided physical and psychological support and advocated for their patient. Caregiving had a substantial impact on the caregiver’s mental wellbeing.

**Conclusions:**

Overall, C3G/primary IC-MPGN presented a substantial burden to patients and caregivers, with a need for timely diagnosis, psychological support for patients and caregivers, improved HCP-patient communication, and treatments which target the cause of C3G/primary IC-MPGN to stabilize the disease.

## 1 Introduction

C3 glomerulopathy (C3G) and primary (idiopathic) immune complex membranoproliferative glomerulonephritis (IC-MPGN) are rare progressive kidney diseases affecting 15−16 in every 100,000 people in Europe (1, 2).

C3G is a rare pattern of injury caused by dysregulation of the alternative complement pathway. Affecting both children and adults, C3G can be genetic or acquired (2, 3). Though clinical course and outcomes remain poorly characterized, around 50% of C3G patients reach kidney failure within 10 years of diagnosis (4–9). While kidney transplantation is an option, C3G recurs in up to 89% of patients (10). IC-MPGN can be either secondary (i.e. caused by immunoglobulin deposited by an underlying illness), or idiopathic (primary; where no underlying illness can be determined) (11). Primary IC-MPGN shares overlapping pathophysiological features with C3G, due to both being the product of C3 dysregulation; however, in IC-MPGN, activation of the classical complement pathway is also involved (2, 12). Similar clinical presentations and outcomes mean a kidney biopsy is needed for a definitive diagnosis (13, 14). Early diagnosis and intervention are thought to be important to slowing progression and improving outcomes in patients with chronic kidney disease (CKD) (15).

While the Kidney Disease Improving Global Outcomes (KDIGO) 2021 clinical practice guidelines offer some treatment guidelines for C3G (16), until recently there were no approved treatments targeting the underlying cause of C3G or primary IC-MPGN (13). Since this study launched, pegcetacoplan and iptacopan have been approved by the United States (US) Food and Drug Administration (FDA) and European Medicines Agency (EMA) to treat adults with C3G. Additionally, pegcetacoplan is also indicated for use in adolescents and to treat primary IC-MPGN (17–20). Pegcetacoplan directly targets C3 and C3b, while iptacopan targets Factor B. Prior to these approvals, existing treatment strategies included supportive measures alone, including immunosuppressants and anti-hypertensives, which had limited efficacy in preventing or delaying disease progression (8, 21–23).

In an evolving treatment landscape, to guide clinical practice, guidelines, and future research, it is important we understand clinicians’ perspectives, and what is important to patients and caregivers when managing their illness. While limited quantitative studies have shown the clinical burden, treatment patterns and profound impact on health-related quality of life (HRQoL) associated with C3G/primary IC-MPGN (24, 25), insights are often limited by the structured nature of responses. Similarly, standardized patient- and caregiver-reported outcome measures often fail to capture disease-specific nuance or what matters to patients and caregivers (26–28). Qualitative studies are necessary to capture the depth of experience and provide the freedom to explore key barriers to timely diagnosis, perceptions of treatment and the unmet needs of patients and caregivers (29). This study aimed to provide this insight using a qualitative interview approach to understand the personal experiences and perspectives of healthcare professionals (HCPs), patients, and informal (voluntary) caregivers. Specifically, we investigate diagnosis, disease burden and management of C3G/primary IC-MPGN to more deeply understand the diagnostic journey, the burden associated with C3G/primary IC-MPGN and its treatments, and to identify any unmet needs in order to inform future research and clinical practice.

## 2 Materials and methods

### 2.1 Design

Data were collected via semi-structured one-to-one interviews with patients with C3G/primary IC-MPGN, their caregivers, and HCPs in France (FR), Germany (DE), Italy (IT), Spain (ES), the United Kingdom (UK), and the United States (US), conducted between July 2024 and January 2025. Demographic data were collected via an online survey. Due to the rare nature of C3G and primary IC-MPGN, all three participant types were selected based on a convenience sample of those who met all eligibility criteria to participate in the study.

HCPs were recruited via data collection agencies in each country, who contacted HCPs on their existing panels. Those eligible to participate had a primary specialty of nephrology or internal medicine (FR and ES only), currently managed at least one patient with a biopsy-confirmed C3G or primary IC-MPGN diagnosis, treated at least one patient with C3G or primary IC-MPGN who wasn’t taking part in a clinical trial, and currently treated patients residing in the US, FR, DE, IT, ES or UK (EU5). There were no exclusion criteria for HCPs.

Patients were eligible to participate if they were aged ≥16 years at data collection, had biopsy confirmed C3G or primary IC-MPGN, and were currently residing in the US, FR, DE, IT, ES or the UK. Patients were excluded if they had previously taken part in a clinical trial relating to treatment of their C3G/primary IC-MPGN, or were currently taking part in any clinical trial, and/or if they had a secondary cause for their C3G/IC-MPGN.

Caregivers were eligible to participate if they were aged ≥18 years and an informal (voluntary) caregiver for a patient that met the patient eligibility criteria (professional caregivers were excluded). Patients and caregivers were recruited via local data collection agencies, who recruited through patients’ treating HCPs, patient advocacy groups, and online networks, including social media.

Both HCPs and patients were asked to complete a survey to gather treatment information, but caregivers were not offered a survey to complete. Instead, caregivers were asked to complete a small number of demographic questions. All participants were asked to complete a semi-structured, one-to-one, one hour interview to gather perceptions and experiences of the diseases. All participants were remunerated for their time at fair market value rates. All participants provided informed consent to participate and have their interviews audio recorded prior to data collection.

### 2.2 Data collection

Interviews were conducted following a short eligibility screening questionnaire and online survey to collect demographic data. Interviews lasted approximately 60 minutes and were conducted via telephone or web-assisted platform in local language by experienced interviewers. Interviewers were trained by the lead qualitative investigator (EH). Interviews were semi-structured, with interviewers following discussion guides. HCP interviews covered: (1) diagnosis of C3G/primary IC-MPGN, (2) barriers to treatment, and (3) HCP perceptions of current treatments and prospects. Patient interviews covered: (1) daily life living with C3G/primary IC-MPGN, (2) onset and diagnosis of C3G/primary IC-MPGN, (3) impact of symptoms on daily life, and (4) C3G/primary IC-MPGN treatment journey since diagnosis. Caregiver interviews covered: (1) daily life caring for a person with C3G/primary IC-MPGN including how they support the person, (2) impact of symptoms and disease progression on the daily life of the person they cared for and disease progression concerns, (3) treatments and quality of care, and (4) impact on the caregiver. All interviews were audio recorded with participant awareness and consent and transcribed verbatim to facilitate data analysis. Interview transcripts were reviewed on receipt for accuracy and dependability.

### 2.3 Analysis

Thematic analysis was conducted by EH, LM, MA, and RN using the six-phase framework set out by Braune and Clarke, 2006 (30), with the steps outlined in **Table 1**. Transcripts were translated by qualified translators from local language into English where necessary (27 transcripts) and checked for accuracy prior to analysis. All transcripts were initially reviewed independently by EH, LM, MA and RN to become familiar with the data and identify preliminary concepts. An initial coding framework was developed based on recurring patterns observed across transcripts. This codebook was then applied to all transcripts using line-by-line coding in ATLAS.ti v25, with MA and RN undertaking primary coding. As coding progressed, the codebook was updated with additional codes as they emerged.

**Table 1:** Thematic analysis steps.

Phase	Description
Phase 1: Familiarization of data	Qualitative researchers (EH, LM, MA, and RN) independently reviewed all transcripts.
Phase 2: Generation of initial codes	Initial codes were discussed by EH, LM, MA, and RN (also aided by the discussion guide), and an initial draft of a codebook was generated, which detailed the code and described how the code should be applied to the interview data. Two researchers (MA and RN) applied the codebook to the dataset by undertaking line-by-line coding of interview transcripts and utilizing ATLAS.ti software (v25.0) to code, organize, and manage the data to facilitate analysis and interpretation. As the coding progressed, the codebook was updated to add additional emerging codes or sub-codes.
Phase 3 and 4: Searching for and reviewing themes	During multiple discussion sessions, EH, LM, MA, and RN searched for themes amongst the data and coding, and by utilizing ‘mind mapping’ techniques themes were reviewed and refined. A qualitative data log captured summaries of discussions and ‘memos’ relating to data, producing an auditable train of the analysis.
Phase 5: Defining the themes	When consensus on themes was reached through discussion between EH, LM, MA, and RN the report was produced.

Once transcripts had been coded, analyst triangulation was conducted, where EH, LM, MA and RN met to discuss each transcript individually, quality check coding and add any further codes not initially identified. During these meetings, coded transcripts were reviewed, discrepancies in coding were discussed and resolved, and agreement was reached on code application and interpretation. Concept saturation was assessed separately for each participant group (healthcare professionals, patients, and caregivers) and was considered achieved when no new codes or concepts were identified in successive interviews within a participant group.

Following completion of coding, related codes were grouped and collated into candidate themes. Themes were reviewed and refined through multiple team discussions, supported by visual ‘mind mapping’ techniques, to explore relationships between themes and ensure internal coherence and distinction between themes. Final themes were agreed by consensus among the analysis team and were clearly defined and named.

### 2.4 Ethics

We carried out this study in line with all relevant national and international guidelines at time of data collection including the declaration of Helsinki. Centralized methodological ethical approval was obtained from the Western Copernicus Group Independent Review Board based in Puyallup, Washington, US. (https://www.wcgirb.com) (WCG Protocol number: 20241694). All participants provided informed consent to participate and have their interviews audio recorded prior to data collection.

## 3 Results

We interviewed forty-two participants; twenty-one HCPs (US: 8, FR: 2, DE: 4, IT: 3, ES: 3, UK: 2), thirteen patients (US: 2, FR: 1, DE: 4, IT: 2, ES: 2, UK: 2), and eight caregivers (US: 1, FR: 0, DE: 4, IT: 1, ES: 2, UK: 0). Caregivers and patients were recruited as linked pairs; their relationships are shown in **Table 2**. HCPs treated a mean (SD) of 12.0 (22.7) patients with C3G/primary IC-MPGN in a typical month. Over three-quarters (77%) had managed patients with C3G/primary IC-MPGN for >10 years, with most (67%) seeing patients in a teaching hospital and in an urban setting (76%; **Table 2**).

**Table 2:** Healthcare provider, patient, and caregiver demographics.

Demographics	US + EU5	US	EU5
HCP demographics			
HCP’s primary medical specialty, n (%)			
Base (All HCPs)	21	8	13
Pediatric nephrologist	2 (10%)	–	2 (15%)
Post-transplant nephrologist	6 (29%)	2 (25%)	4 (31%)
Dialysis nephrologist	3 (14%)	1 (12%)	2 (15%)
Other type of nephrologist	10 (48%)	5 (62%)	5 (38%)
Number of patients with C3G/primary IC-MPGN currently treated in a typical month			
Mean (SD)	12.0 (22.6)	22.6 (35.2)	5.5 (3.3)
Number of years HCP has treated patients with C3G/primary IC-MPGN, n (%)			
1–4 years	3 (14%)	–	3 (23%)
5 to 10 years	2 (10%)	–	2 (15%)
11 to 15 years	6 (29%)	2 (25%)	4 (31%)
16 to 20 years	5 (24%)	3 (38%)	2 (15%)
21 or more years	5 (24%)	3 (38%)	2 (15%)
Primary setting in which HCP sees patients with C3G/primary IC-MPGN, n (%)			
Specialist treatment center	3 (14%)	1 (12%)	2 (15%)
Teaching hospital	14 (67%)	3 (38%)	11 (85%)
Non-teaching hospital	2 (10%)	2 (25%)	–
Ambulatory care	2 (10%)	2 (25%)	–
Primary setting HCP sees patients with C3G/primary IC-MPGN, n (%)			
Urban	16 (76%)	4 (50%)	12 (92%)
Suburban	4 (19%)	3 (38%)	1 (8%)
Rural	1 (5%)	1 (12%)	–
Patient demographics			
Patient age			
Base (All patients)	13	2	11
Years, Mean (SD)	41.8 (14.5)	46.5 (6.4)	40.9 (15.6)
Patient sex, n (%)			
Female	11 (85%)	2 (100%)	9 (82%)
Male	2 (15%)	–	2 (18%)
Patient ethnicity, n (%)			
Base (All patients apart from France)*	12	2	10
White	10 (83%)	1 (50%)	9 (90%)
Other	1 (8%)	–	1 (10%)
Prefer not to say	1 (8%)	1 (50%)	–
Patient current employment status, n (%)			
Working full time	5 (38%)	1 (50%)	4 (36%)
Working part time	2 (15%)	–	2 (18%)
Student	2 (15%)	–	2 (18%)
Unemployed	1 (8%)	–	1 (9%)
On long term sick leave	1 (8%)	–	1 (9%)
Retired	1 (8%)	–	1 (9%)
Prefer not to say	1 (8%)	1 (50%)	–
Biopsy confirmed diagnosis of C3G or primary IC-MPGN, n (%)			
C3G	8 (62%)	2 (100%)	6 (55%)
primary IC-MPGN	5 (38%)	–	5 (45%)
Patient sub-type of C3G, n (%)			
Base (All patients with C3G)	8	2	6
DDD	1 (12%)	–	1 (17%)
C3GN	4 (50%)	1 (50%)	3 (50%)
Don’t know/my C3G has not been classified	3 (38%)	1 (50%)	2 (33%)
If patient had a kidney transplant, n (%)			
Yes	3 (23%)	1 (50%)	2 (18%)
No	10 (77%)	1 (50%)	9 (82%)
If patient was receiving dialysis**, n (%)			
Yes	3 (23%)	1 (50%)	2 (18%)
No	10 (77%)	1 (50%)	9 (82%)
If the patient had a caregiver to support them in their everyday tasks, n (%)			
Yes	7 (54%)	2 (100%)	5 (45%)
No	6 (46%)	–	6 (55%)
Time since C3G diagnosis, n (%)			
Less than 1 year ago	1 (8%)	–	1 (9%)
1–4 years ago	4 (31%)	–	4 (36%)
5–9 years ago	5 (38%)	1 (50%)	4 (36%)
10 years ago or more	3 (23%)	1 (50%)	2 (18%)
Treatments received to help manage C3G/primary IC-MPGN, n (%)			
Eculizumab	1 (8%)	–	1 (9%)
Anti-hypertensives	6 (46%)	1 (50%)	5 (45%)
Corticosteroids	7 (54%)	2 (100%)	5 (45%)
Mycophenolate mofetil	4 (31%)	1 (50%)	3 (27%)
Investigational therapy	1 (8%)	–	1 (9%)
Other	1 (8%)	–	1 (9%)
None	1 (8%)	–	1 (9%)
Patient living status, n (%)			
I live alone in my own home or rented property	1 (8%)	–	1 (9%)
I live with my partner/spouse/children in our own home or rented property	7 (54%)	1 (50%)	6 (55%)
I live with other family or friends in our own home or rented property	3 (23%)	1 (50%)	2 (18%)
Other	2 (15%)	–	2 (18%)
Caregiver demographics			
Caregiver age			
Base (All caregivers)	8	1	7
Years, Mean (SD)	49.9 (11.7)	55.0 (-)	49.1 (12.4)
Caregiver sex at birth, n (%)			
Female	4 (50%)	1 (100%)	3 (43%)
Male	4 (50%)	–	4 (57%)
Caregiver ethnicity, n (%)			
White	7 (88%)	–	7 (100%)
Prefer not to say	1 (12%)	1 (100%)	–
Caregiver relationship to patient, n (%)			
Spouse/partner	3 (38%)	–	3 (43%)
Parent	1 (13%)	–	1 (14%)
Sibling	1 (13%)	–	1 (14%)
Other relative	2 (25%)	1 (100%)	1 (14%)
Friend	1 (13%)	–	1 (14%)

US, United States; EU5, European 5; HCP, healthcare professional; C3G, Complement 3 Glomerulopathy; IC-MPGN, Immune Complex Membranoproliferative Glomerulonephritis; SD, standard deviation; DDD, dense deposit disease; C3GN, C3 glomerulonephritis.

*Patient ethnicity was not allowed to be asked in France due to the French Constitutional Council prohibiting the collection of this information. ** n=3 other patients had previously received dialysis.

Mean (SD) patients age was 41.8 (14.5) years (age range 20-64), eleven of thirteen were female, and ten of twelve were white (see notes under **Table 2**). Five of the thirteen patients were working full-time, and two part-time. In all, eight of the thirteen patients were diagnosed with C3G and five of thirteen with primary IC-MPGN. Of those diagnosed with C3G, four of the eight patients had C3 glomerulonephritis, one of the eight had dense deposit disease, and disease type was unknown in the remaining three. Of all patients, twelve of thirteen were diagnosed with C3G/primary IC-MPGN more than a year prior to interview, with eight of thirteen patients diagnosed >5 years prior. Three of thirteen patients had received a kidney transplant, and three of thirteen were on dialysis (one of whom had previously received a kidney transplant). The most common treatments received were corticosteroids (received by seven of thirteen patients), anti-hypertensives (six of thirteen patients), or mycophenolate mofetil (four of thirteen patients). The majority (seven of thirteen) of patients were living at home with a partner/spouse/children. In all, seven of the thirteen patients reported needing a voluntary caregiver. Mean (SD) age of caregivers was 49.9 (11.7) years, four of the eight caregivers were female, and seven described themselves as White (**Table 2**). Three of eight caregivers were partners or spouses (**Table 2**), and four of the eight caregivers lived with the patient. Select patient clinical characteristics by country can be found in **Table 3**.

**Table 3:** Patient clinical characteristics by country.

Clinical characteristics	US + EU5	US	EU5	FR	DE	IT	ES	UK
Biopsy confirmed diagnosis of C3G or primary IC-MPGN, n (%)								
Base (All patients)	13	2	11	1	4	2	2	2
C3G	8 (62%)	2 (100%)	6 (55%)	1 (100%)	3 (75%)	2 (100%)	–	–
primary IC-MPGN	5 (38%)	–	5 (45%)	–	1 (25%)	–	2 (100%)	2 (100%)
Patient sub-type of C3G, n (%)								
Base (all patients with C3G)	8	2	6	1	3	2	-	-
DDD	1 (12%)	–	1 (17%)	–	1 (33%)	–	–	–
C3GN	4 (50%)	1 (50%)	3 (50%)	1 (100%)	–	2 (100%)	–	–
Don’t know/my C3G has not been classified	3 (38%)	1 (50%)	2 (33%)	–	2 (67%)	–	–	–
If patient had a kidney transplant, n (%)								
Yes	3 (23%)	1 (50%)	2 (18%)	–	–	1 (50%)	–	1 (50%)
No	10 (77%)	1 (50%)	9 (82%)	1 (100%)	4 (100%)	1 (50%)	2 (100%)	1 (50%)
If patient was receiving dialysis*, n (%)								
Yes	3 (23%)	1 (50%)	2 (18%)	–	1 (25%)	1 (50%)	–	–
No	10 (77%)	1 (50%)	9 (82%)	1 (100%)	3 (75%)	1 (50%)	2 (100%)	2 (100%)

US, United States; EU5, European 5; C3G, Complement 3 Glomerulopathy; IC-MPGN, Immune Complex Membranoproliferative Glomerulonephritis; DDD, dense deposit disease; C3GN, C3 glomerulonephritis.

* n=3 other patients had previously received dialysis.

We identified three key themes from HCP data, nine from patient data, and two from caregiver data. Themes are illustrated with salient quotes extracted from interview data. Quotes have been edited for concision, to remove repetition, and to improve readability while maintaining context. Edits are indicated by [ … ].

### 3.1 HCP interview themes

#### 3.1.1 Theme 1: patients with ‘milder’ or asymptomatic disease experienced long delays to accessing specialist care and diagnosis, while acute or moderate-severe patients typically received a timely diagnosis

All twenty-one HCPs identified a need for timely diagnosis of C3G/primary IC-MPGN. Eighteen of the twenty-one HCPs indicated ‘milder’ or asymptomatic patients were at greatest risk of misdiagnosis and delayed referral to specialist care, which was detrimental to their overall prognosis.

*“It can be a smoldering burn, but it’s not a bonfire. Those people who have a bonfire going on in those kidneys, they get picked up. It’s the burn, the smoldering disease, that is slowly eating away the kidneys, those people get missed.” HCP, [US].*

Eight of twenty-one HCPs recognized a failure to identify and investigate non-specific symptoms (e.g. proteinuria) as potential symptoms of rare disease in primary care which delayed timely diagnosis. HCPs also stated that patients often delayed visiting primary care due to non-specific symptoms or not recognizing they were seriously ill.

*“Another thing or factor is the GP, who needs to recognize it. And not every GP is trained in nephrology, which means that they first have to come up with the idea that it might be kidney disease. This may take some time.” HCP, [DE].*

*“You neglect the symptom, which can be edema, non-drinker, minimal hematuria, so the color, [ … ] of the urine. This may be neglected more by the family or the patient than by the general practitioner [ … ]” HCP, [IT].*

Once in specialist care, six of twenty-one HCPs perceived patients were reluctant to undergo biopsy as they did not understand why a biopsy was necessary, with three of these six noting that this was particularly prevalent among younger patients.

*“Sometimes patients, they may not understand exactly why they need a biopsy, and so convincing them about the need for biopsy is actually one of the big factors.” HCP, [US].*

*“Younger patients may take a longer time to make a decision about whether they need a biopsy. I would say middle-aged to older patients, they’re pretty clear that they have a problem and most of them have the insight.” HCP, [US].*

In contrast, patients presenting with acute or moderate-severe symptoms were referred rapidly to a nephrology service because they presented to an emergency or urgent care facility with more apparent symptoms.

*“More often we see them from the emergency room [ … ] either given the acute symptoms they are hospitalized, or they are hooked up to the DH outpatient service, in a more or less short time.” HCP, [IT].*

#### 3.1.2 Theme 2: once the need for a specialist was recognized, availability of specialist nephrologists experienced in treating the condition became a barrier to treatment

Even when referred to a specialist, ten of the twenty-one HCPs interviewed noted a lack of specialist nephrologists trained in treating C3G/primary IC-MPGN and appointment availability. Seven of the Eight of HCPs interviewed in the US stated that HCPs tended to be based in larger cities, with long travel times presenting a barrier to rural or semi-rural patients.

*“Well, there aren’t many. In the UK, there are two centers that do this, who specialize in these sorts of diseases” HCP, [UK].*

*“Depending on where you live, nephrology is very sparse. If you’re not near a major city or in the suburbs of a major city and you’re in rural America, there are not a lot of specialists in anything, and you might be traveling two or three hours to get to a specialist.” HCP, [US].*

#### 3.1.3 Theme 3: HCPs perceived that there were limited treatment options available for patients at the time of interview, but were hopeful for future treatment options

When finally treated for C3G/primary IC-MPGN, twelve of twenty-one HCPs highlighted a lack of effective treatments available, with current treatments aiming to maintain kidney function for as long as possible. Twelve of the twenty-one HCPs, however, were excited for future treatment options targeting the complement cascade.

*“Although we already have some immunosuppressants, we need specific therapies that block complement for this condition” HCP, [ES].*

*“Moderator: What is currently the greatest unmet need in your patients?*

*HCP: Efficacy. I still think that I can get about 25 to 30% of my patients into remission, but that leaves two-thirds of my patients hanging out there with either partial or no remission at all, and they progress to kidney failure or transplant in the span of three to five years, maybe longer, but they eventually attain end-stage kidney disease.” HCP, [US].*

### 3.2 Patient interview themes –diagnosis

#### 3.2.1 Theme 1: the length of time to receiving a confirmed diagnosis was highly variable

Eight of the thirteen patients interviewed reported diagnostic delays due to testing, a lack of recognition of their illness, or misdiagnoses due to non-specific symptoms. One of the thirteen patients initially consulted a cardiologist as non-specific symptoms made them suspect a heart problem, leading to delays in receiving a biopsy. Meanwhile, one patient in the UK reported long monitoring periods prior to diagnosis due to milder symptoms. Once symptoms became acute, they were rapidly referred to a nephrologist for a biopsy.

“*My GP who ran a series of blood tests. And they said, “[ … ] your kidney function really doesn’t look right, and you’ve got really high blood pressure [ … ] they said they’re gonna monitor me every two months [ … ] for about six months. And in that period, I started to feel just really, really tired [ … ] after one of these routine blood tests [ … ] I got an urgent call saying, “You’ve gotta get in here now. [ … ] your kidney function’s really got very low.” So, [ … ] the next day they sent me up to the hospital. [ … ] I think it was only about two or three days, they sent me for a biopsy.” PAT, [F, 53, UK].*

Initial misdiagnosis was reported by four of thirteen patients, one of whom was misdiagnosed by three different medical professionals. These patients felt that misdiagnoses had increased their suffering.

*“[ … ] I started having weird urine and I was not feeling well and I went to my PCP [primary care physician] and they were like, yes, there is some blood in your urine, but have you been on your period? And I am like, yes. [ … ] so he really did not think much about it. [ … ] and then I went to my OBGYN who was like, okay, it is time to check for some endometriosis [ … ] I have had that happen before. Had a couple of laparoscopic. So we did another one. And then in the pre-work check, [ … ] that is where they saw the kidney function was off, and that is when they sent me to a nephrologist.” PAT, [F, 42, US].*

*“[ … ] I think that if I had been diagnosed with the disease from the beginning, I might not have gone through so many years of suffering. It was an ordeal.” PAT, [M, 53, ES].*

Five of the thirteen patients had a relatively quick diagnosis, with four of these five presenting with acute symptoms which were ‘visible’ (either physically or clinically).

### 3.3 Patient interview themes – symptom burden

#### 3.3.1 Theme 1: patients experienced physical exhaustion, weakness, and pain from their condition, impacting their ability to take part in activities which required physical energy

Though often non-specific, symptom burden was high. Eight of the thirteen patients noted that they experienced highly burdensome fatigue/exhaustion, which manifested both physically and mentally. Fatigue/exhaustion due to illness and treatment left them physically weak and feeling drained which impacted work, leisure and social activities, with physical pain adding to activity impairment. Four of the thirteen patients indicated that being unable to keep up with hobbies and physical activities exerted a mental toll, leaving them feeling not “like before” or experiencing “some sadness”.

*“[ … ] the feeling that you are completely exhausted and just feel weak, so really drained and low on energy … you no longer have the physical strength, you feel as though you’re being drained of energy” PAT, [F, 47, DE].*

*“Yes, because while I was, um, undergoing treatment, the doctor did tell me, look, [ … ] you have to make changes [ … ] I recommend that you stop the sports activity, which was something that I loved … I am, well, a musician too and I could not stand for a long time, so, I could not work or do that activity that I like so much [ … ] I did stop the activity, which caused me some sadness. I couldn’t play football, work … it was a lot of emotions” PAT, [M, 53, ES].*

#### 3.3.2 Theme 2: patients experienced fatigue as mental and cognitive exhaustion, which impacted their ability to take part in activities which required cognitive concentration

Beyond the physical, patients also reported experiencing mental and cognitive exhaustion, which manifested as being *“much less attentive, much less efficient as well” PAT, [F, 29, FR]*. This affected many aspects of their daily lives including limiting work productivity, with some patients having to reduce their working hours, and leisure time.

*“… tiredness … that’s the main thing this is why I work part time*” *PAT, [F, 53, UK]*.

*“leisure time is then somewhat limited in the afternoon” PAT, [F, 51, DE].*

*“I have to refuse certain things, where I can’t do what’s on offer such as outdoor activities” PAT, [F, 29, FR].*

Physical and mental exhaustion ultimately led to patients feeling unfulfilled and less joyful, with feelings of disappointment and guilt that they could no longer do the activities they did previously.

“*I feel like I’m not thinking as fast as I usually would, it’s not very nice … I’m less able to concentrate and so inevitably, in my previous joy, it always had an impact on what I was doing” PAT, [F, 29, FR].*

“*I understand somewhere that maybe some members of the family are a bit disappointed that we don’t have the crazy times that we used to anymore” PAT, [F, 47, DE].*

Patients also reported that sleep disturbance further impacted their mental and cognitive abilities, causing feelings of irritability and restlessness.

“*I tend not to sleep very well, so that doesn’t help either … I can’t concentrate*” *PAT, [F, 29, FR]’*.

“*My brain works less well, I’m much more irritable” PAT, [F, 29, FR].*

#### 3.3.3 Theme 3: other symptoms frequently cited as highly burdensome were swelling and water retention

Other highly burdensome symptoms identified included swelling (reported by six of thirteen patients) and water retention (reported by four of thirteen patients), which further limited their capacity to do everyday tasks.

*“I would say that the swelling one of the biggest ones [ … ] before I had all of this going on, I was a very active person. I did dance competition teams at university. I was going to the gym 6 days a week. But because of the fluid, even if I take the tablets, if I stand on my feet for too long, stand for more than 15 minutes, my feet and legs start burning and then swelling, then I start getting kidney pain.” PAT, [F, 21, UK].*

#### 3.3.4 Theme 4: patients frequently expressed fears for the future, which contributed to the substantial emotional burden of disease. depending on their circumstances, patients either feared that their condition would worsen over time or that it would return post-transplant

Beyond symptoms, fear associated with illness also represented a substantial burden. Patients frequently expressed uncertainty, anxiety and fear around their diagnosis and living with C3G/primary IC-MPGN. Pre-transplant patients’ were concerned about worsening of their condition, decreasing effectiveness of treatment options available, and complications (due to their condition or transplant). Post-transplant patients feared that their condition would return.

*“The only thing that worries me is that [ … ] they tell me that it is chronic and that there is no cure. That worries me, even though I feel good. [ … ] it will never go away. It’s like having to handle it constantly. [ … ] sometimes, I don’t know if I’m okay today and tomorrow, because of the stress or problems, my proteinuria can skyrocket to alarming levels, and I don’t always have medical care available” PAT, [M, 53, ES].*

*“The big thing with this disease is, there’s a high degree of chance that disease can return with a transplant. And that’s what they were most worried about.” PAT, [F, 53, UK].*

Anxieties about the future represented a significant mental burden to patients with one of the thirteen patients reporting needing therapy due to their anxiety post-transplant.

“*When I texted my therapist that I was having a kidney transplant, she is like, okay, I will get you in as soon as I can when you are able. [ … ] so while I was still recuperating in the city where I had transplant, I was having therapy.” PAT, [F, 42, US]*.

### 3.4 Patient interview themes – treatment

#### 3.4.1 Theme 1: pre-dialysis and pre-transplant patients received a variety of medications as treatments, and some cited frustration with systemic barriers to receiving advanced or investigational treatments

Effective treatment is key to controlling symptoms, however patients reported substantial systemic barriers to accessing more advanced treatments. Prior to dialysis and transplant, patients reported receiving a variety of treatments likely reflecting the diversity of treatment settings and disease stages in our sample. While patients were aware of new/advanced treatment, they reported systemic barriers to receiving these, including insurance provisions, overall cost, and current treatment guidelines, which meant they could not access advanced or investigational treatment until their condition had worsened.

“*… the doctor has already said that there is probably a completely new immune drug coming soon. But this is quite expensive and that would really only be allowed for people whose kidney values are really getting a lot worse, but before you would have to go to dialysis. But I’m probably still too healthy and that’s why they haven’t told me more about how to take it, or whether it’s injected, or whether you have to go to the hospital. I don’t know anything about that yet.” PAT, [F, 47, DE].*

#### 3.4.2 Theme 2: in patients who were receiving dialysis, effective symptom relief was experienced initially, however treatment was highly burdensome and the treatment schedule untenable long-term

Treatments often added to patient burden. In this sample, three patients were currently receiving dialysis while three others had received dialysis previously. Prior to dialysis, patients reported that they felt extremely unwell with two of these six patients noting that their disease was very severe at diagnosis and they were offered dialysis within a few months. Those that had received dialysis felt it offered initial effective symptom relief due to the severity of their condition, however, the schedule commitment was highly burdensome, and untenable long-term.

*“I would say, before dialysis, I was a wreck. I was exhausted. I had no energy. I had … like my legs were swollen and could be … I was uncomfortable and that dialysis was needed at that point. I felt symptoms, and dialysis had corrected, but that’s not something I feel. The symptoms that bother me now aren’t necessarily a symptom of it. It’s more of a mental … what you call … my commitment” PAT, [F, 51, US].*

Patients highlighted that dialysis was time-consuming, expensive (especially if travelling away from home) and affected many aspects of their everyday lives due to organizational requirements and the fatigue caused afterwards. Patients felt as though their lives were “*controlled and restricted … I feel jailed by it … I don’t have choice … I just feel trapped*” *PAT, [F, 51, US]* by dialysis.

“*the minimum is three hours, three times a week, so that’s nine hours a week where I’m not available at all*” *PAT, [F, 51, DE].*

“*It cannot be done for free all over the world … it is therapy that costs a lot of money. To do it 3 times a week, it costs you even more*” *PAT, [F, 20, IT].*

“*I’m wiped out” PAT, [F, 51, US].*

“*I felt really tired and devastated*” *PAT, [F, 20, IT].*

#### 3.4.3 Theme 3: visible manifestations of disease or treatment side effects (e.g. weight gain) detrimentally impacted patient self-image or their relationships with others

In addition to the direct burden of C3G/primary IC-MPGN, side-effects associated with treatments such as steroids also exerted a burden. Four of thirteen patients reported that weight gain and swelling negatively impacted their perceptions of self and relationships with others. *PAT, [F, 21, UK]* expressed that their weight gain affected their relationship with their partner causing *“very bad self-esteem issues”* as they felt they did not look how they did when they first met their partner. *PAT, [F, 21, UK]* also felt weight gain added strain to their relationship with their mother as *“she would always comment on all the weight I’ve gained”. PAT, [M, 53, ES]* expressed that *“emotionally it [weight gain] was awful”* especially as their work as a musician involved them being on stage in front of people which made them feel *“exposed”*.

#### 3.4.4 Theme 4: reaching a state of disease stability was an achievable and highly important marker of treatment success from the patients’ perspective

When asked about key treatment goals, nine of the thirteen patients said achieving disease stability was a key marker of treatment success with one patient reasoning *“Disease stability is critical, because, I mean if you’re not stabilized, then it’s out of control, and I’ll be looking for a kidney … treatment success makes me stable, makes me able to live my life” PAT, [F, 51, US].* For patients, disease stability meant controlling their C3G/primary IC-MPGN through active management, halting disease progression and maintaining a constant state. Among younger patients, no longer experiencing symptoms and feeling well was another indicator of success, highlighting the impact of high symptom and side-effect burden.

*“At age 23, success means that I can feel well, that I no longer have the symptoms that I had before, and well, for someone my age, the aesthetic level, the aesthetic side, has a strong impact. There was a time when I struggled to accept myself as I was. So, given the improvements both in how I feel, and aesthetically, that for me is my success, the fact that I feel well … I was not prevented from doing anything” PAT, [F, 23, IT].*

Whilst stability was important, four of thirteen patients also reported that they hoped for a “cure”, however they accepted stability as a key outcome, with one patient remarking that it is “*much better than it being worse … it’s bittersweet … it’s being managed, and I’m reacting well to the treatment, where some people don’t react at all” PAT, [F, 21, UK].*

### 3.5 Caregiver interview themes

#### 3.5.1 Theme 1: caregivers perceived that fatigue was a particularly bothersome symptom for patients and that treatment options were limited

Burden of illness is not just felt by patients, but by caregivers who must understand and share this burden. It is important that caregivers can identify sources of burden to help manage them. Five of the eight caregivers identified that the person they cared for experienced highly burdensome fatigue and exhaustion, which impacted their ability to complete physical and mental tasks. In addition, two of the eight caregivers also reported a loss of appetite in the person they cared for, with one reporting extreme weight loss. A further two of the eight caregivers reported that blood pressure issues and water retention were key symptoms, with one reporting that water retention caused a loss of sleep in the person they cared for.

“*If you have these kinds of kidney problems, then you’re just not as efficient anymore and you just don’t have the stamina for certain things like running, taking care of the garden or something*.” *CG, [M, 67, DE]*.

“*Yes, she also complained that she had little appetite, and the weight loss [ … ] she hardly wanted to eat either.*” *CG, [M, 61, DE].*

*“So water retention is what definitely keeps them up at night [ … ]. She felt constricted even in bathing slippers or flip-flops. [ … ] that was also a circumstance in which she first had to get used to developing a routine again and an idea of how to do it in the summer, because [ … ] it also hurt a lot.” CG, [M, 40, DE].*

Beyond the physical, caregivers also reported a significant mental burden of disease, with those they cared for becoming introverted, irritable, unmotivated and embarrassed due to their condition.

“*And what also happens is that she has suffered a relatively strong decline in her mental health recently … psychologically, actually … but now it’s hit her mental health in that she’s lost the will to live a bit.” CG, [F, 45, DE]*.

*“I would say irritability … I would say it’s like a moody, unpredictable person … he’s definitely become introverted and uninterested”. CG, [F, 55, US]*.

Most caregivers identified that current treatment options were limited, with four of the eight caregivers stating that they knew kidney transplant was the only cure.

*“I mean, you can have great people, but they can’t fix the problem unless he gets like, you know, a kidney transplant or something, but that’s not even on the radar at this second.” CG, [F, 55, US].*

*“That maybe there is still a need to do more research to attack, that is, to completely remove the disease, because I believe that maybe it will have a cure, but the accurate treatment has not yet come out, but at least this one is a little more stable.” CG, [F, 35, ES].*

#### 3.5.2 Theme 2: caregivers provided multifaceted care and support for patients, which had both a pragmatic and emotional impact on caregivers

Caregivers played many roles to support patients and help with the burden of illness. All eight caregivers interviewed described themselves as providing support on pragmatic day-to-day tasks e.g. cooking and cleaning, shopping, taking the patient to and from the hospital for appointments including dialysis, picking up medications from the pharmacy and reminding the patient to take their medications. Six of the eight caregivers mentioned that they also provided psychological, motivational, and emotional support to cheer up the patient, nurturing a sense of normality and reminding them that life goes on beyond their illness. Beyond directly supporting the patient, five out of the eight caregivers reported acting as an advocate, ensuring doctors followed up their care appropriately and writing appeals to welfare officers.

*“I was mainly responsible for shopping here or going to the nearest hospital for dialysis. Those were just our tasks and areas of responsibility … and she herself couldn’t do everything independently, everyday.” CG, [M, 61, DE].*

*“Well, of course, personal support is important as well, so you might say psychologically. Just to be there for him and to cheer him up again, to remind him that life goes on and, well, get the drugs and things like that from the pharmacy.” CG, [M, 67, DE].*

*“I’d say I have been a fighter, so to speak … I had to go in person to talk, to say, to get her hospitalized … you have to roll up your sleeves and always go there. Talk, talk and then they listen to you”. CG, [F, 57, IT].*

These caregiving duties had both a practical and emotional impact on their lives. Caregivers reported a lack of free time for their own hobbies, interests, and life tasks and that caregiving impacted their work lives, requiring additional flexibility from their employer. They also noted caregiving had a substantial emotional impact, with caregivers feeling uncertainty and fear for their own future and for their family. One of the eight caregivers reported needing to seek psychological support.

*“… a huge time commitment. This just means that sometimes you have to leave your own things at home a little longer and sometimes you have to let your private life go a little bit. You’re always on call, and sometimes it’s quite exhausting.” CG, [F, 45, DE].*

“*If you then ask if you can go earlier or if you could have some time off, it’ll be noticed … my immediate line managers know about this and are so usually relatively accommodating when it comes to this*.” *CG, [F, 45, DE].*

*“It was psychologically hard for me, because it was something new, I had little children, and well, thank goodness I had the support of my mother too … at first I had to go to the psychologist myself because I was having to see a person in that state, being about to die … I think that beyond all, seeing a loved one in those conditions is a difficult thing and even more when you have children with that person and that person maybe cannot do everyday activities … I had about two months ago a stress collapse…” CG, [F, 35, ES].*

Despite the negative impact of caregiving, one of the eight caregivers stated that caring had strengthened their bond with their partner, with another reporting that helping their partner maintain their diet had led to a healthier diet for themselves.

*“Of course, it’s an additional burden, but it has not negatively affected us now. I think it’s brought us even closer together. My wife knows she can rely on me even more. I think it has a positive effect on circumstances that it’s not always easy.” CG, [M, 40, DE].*

*“It is not something that I am forced to do, that I see it as a martyrdom of making food, because in the end I also eat well and my children too.” CG, [F, 35, ES].*

## 4 Discussion

Our study provides detailed insight into the personal experiences and opinions of HCPs, patients, and caregivers, using firsthand accounts to highlight the most burdensome aspects of C3G/primary IC-MPGN and unmet needs in treatment and diagnosis. We also highlight the diverse physical and mental support role played by caregivers of patients with C3G/primary IC-MPGN and the impact this has on the caregiver. The high physical and psychological burden to patients observed here supports findings from studies in C3G/primary IC-MPGN, which highlighted the profound impact that disease has on HRQoL (24, 25, 31).

KDIGO have previously highlighted the importance of timely diagnosis of CKD in improving clinical outcomes and delaying kidney failure (15). Here HCPs, patients, and caregivers noted that delays in diagnosis and accessing specialist care were a key problem, particularly among those with milder symptoms (e.g., fatigue, low level hematuria, or edema). Conversely, HCPs and patients both noted that patients who presented with acute or severe symptoms were referred rapidly to a specialist for biopsy. Diagnostic delays and misdiagnosis were attributed to a lack of knowledge of C3G/primary IC-MPGN among primary care physicians, due to symptoms overlapping with other diseases. A survey of primary care physicians found that they were often poorly prepared to identify patients with rare diseases, underestimating their prevalence and lacking confidence in their ability to diagnose them (32). Recent publications have highlighted a need for a standardized and comprehensive diagnostic approach to identifying patients in need of an urgent biopsy (14, 15, 33, 34).

Physicians reported that delays in diagnosis were also attributable to reluctance to have a biopsy, with patients often not understanding why a biopsy was necessary or delaying due to fears. Anxiety is common amongst patients receiving biopsies across a range of disease areas, due to the procedure being invasive and painful. Increased patient knowledge has been shown to alleviate this anxiety to a degree (35–38). When discussing negative experiences in treatment and care, some caregivers highlighted that communication challenges were a problem, demonstrating a need for improved communication between patients, caregivers and physicians (39).

Patients and caregivers both identified that fatigue/exhaustion were amongst the most common and burdensome symptoms for patients; a common theme in C3G, primary IC-MPGN, and across other CKDs (40–42). The standard of care at time of study focused on supportive care to treat symptoms (33). Fatigue/exhaustion, however, are difficult to manage effectively, particularly as patients reported that current treatments contributed to fatigue/exhaustion. In CKD, fatigue experienced by patients was often underdiscussed, with more than half of patients who reported fatigue receiving no advice or treatment for it (43). In a recent survey, only 51% of physicians reported that primary IC-MPGN was a major problem in a patient’s life, and 38% that it impacted their patient’s ability to work/study (31). This suggests a need to improve HCP recognition of the physical impact of C3G/primary IC-MPGN and communication surrounding fatigue (44).

Patients and caregivers also faced a high psychological burden associated with C3G/primary IC-MPGN, treatment, and side effects, with patients reporting sadness, irritability, and poor self-image as well as anxiety and fears over their future. A recent study found nearly three quarters of patients reported experiencing anxiety/depression on the EQ-5D (42). Though data is lacking in C3G/primary IC-MPGN, psychological disorders, such as anxiety and depression, are underdiagnosed in CKD (45). Only 55% of physicians reported that primary IC-MPGN caused emotional upset in patients in a recent survey (31), further demonstrating a need for improved communication regarding the psychological impact of C3G/primary IC-MPGN. The above suggests that psychological support should form a key pillar of patient care to alleviate burden and improve HRQoL (24).

HCPs, patients, and caregivers indicated that current treatment options which targeted symptoms were highly burdensome. Side effects of treatment such as weight gain had a profound impact on self-image and mental health. Patients indicated that dialysis was particularly burdensome due to it being time consuming, expensive, and lifestyle restricting. Patients on dialysis across CKD often have worse HRQoL than those not on dialysis (46, 47), with high levels of anxiety and depression associated with dialysis not just in patients but in their caregivers, too (48–50). Though patients found dialysis to be effective at treating symptoms it does not prevent disease progression. One of the key goals of treatment identified by patients was to achieve ‘stable disease’, which they understood to mean halting disease progression. Novel, less burdensome disease modifying treatments that target the root cause of C3G/primary IC-MPGN are needed to improve patient outcomes and alleviate treatment burden in both patients and caregivers.

Finally, our study provided valuable insight into the caregivers’ perspective of C3G/primary IC-MPGN and the burden faced by them. The role of caregivers was diverse, not only providing physical support but also psychological support, while also acting as an advocate for the person they cared for. Caregivers had to make significant sacrifices to care for patients, sacrificing time for hobbies and interests and disrupting their work hours. When asked, caregivers reported caregiving had a profound impact on their own mental wellbeing, expressing concerns about the uncertainty surrounding disease course and fears for the future. This was explored with the aid of specific questions in the caregiver discussion guide that aided in probing the caregiver about the impact caring had on their mental health. This demonstrates that C3G/primary IC-MPGN has an impact beyond just that experienced directly by patients and that removing the uncertainty surrounding illness via treatment could improve caregiver wellbeing. Psychological support for caregivers as well as patients could help to alleviate some of the burden felt by caregivers.

Ultimately, our findings highlight a need to improve diagnostic processes, access to specialist care and advanced treatments, and for a more holistic and tailored approach to care that includes both patient and caregiver. Clear guidance and education to help primary care physicians recognize symptoms of rare disease and when to send patients for biopsy could reduce diagnostic delay and improve patient outcomes. While organizational interventions have shown success in improving specialist referral waiting times, novel ways of increasing access to specialist care are needed (51). Improved communication between physicians and patients is needed to alleviate patient anxieties surrounding biopsy and to ensure symptoms that are most burdensome to patients are included in treatment decisions. Shared decision making has shown some success at improving knowledge-exchange, involving patients in treatment decisions to ensure they are fully informed of care options and associated risks and allowing inclusion of both clinical factors and patient preferences (39, 44). Limited treatment options mean new treatment options and greater access to advanced treatments are needed to improve symptom management and outcomes. A key marker of treatment success for patients was disease stability. Recently approved complement inhibitor treatments could be key to achieving stability. Both pegcetacoplan and iptacopan showed a significant reduction in proteinuria, with pegcetacoplan also improving eGFR stabilization compared to placebo in phase III trials (52). Reducing proteinuria can decrease the risk of kidney failure and improve outcomes in C3G/IC-MPGN (9). Increased proteinuria and lower eGFR have also been linked to pain, fatigue and poorer HRQoL in immunoglobulin A nephropathy, another rare glomerular disease (53). The above indicates that newly approved treatments for C3G/IC-MPGN could help manage symptoms and reduce burden of illness, though further research is needed. To fully support patients, a more holistic approach is needed, one that includes the psychological impact of C3G/primary IC-MPGN, not just for patient but for caregiver. Psychological support may not just improve HRQoL, but also clinical outcomes. HRQoL has been associated with risk of progression in CKD, and social-psychological support can improve fatigue management in end-stage renal disease (54, 55).

### 4.1 Limitations

For the purposes of this study, we recruited a pragmatic sample designed to recruit a broad range of experiences to produce a rich in-depth dataset, however, this method has limitations. As described in the social science literature, n=12 interviews are typically posited as an adequate number of interviews to achieve “saturation” of concepts, meaning no new themes are uncovered in interviews (56, 57). Though we achieved this globally for HCPs and patients it should be noted that we did not achieve this for caregivers. Therefore, concept saturation may not have been achieved, and some themes may not have been captured that could have influenced our results and discussion. Furthermore, we recruited our sample using convenience sampling due to the rare nature of C3G/primary IC-MPGN, but the overall sample sizes remained relatively small, especially for caregivers. Interpretations from our results therefore should be treated with caution, as our sample may not be reflective of the entire C3G/primary IC-MPGN population. It should be noted that most of the patient sample were female and of white ethnicity, and as we recruited through HCPs and patient advocacy groups there is the potential for selection bias as patients may already have improved HRQoL or disease knowledge due to their interaction with an HCP or patient advocate. This may also have an impact on the generalizability of the results – describing only a certain demographic may present a gap in describing the wider population of these patients and caregivers. While difficult to avoid, this bias was mitigated by also recruiting from other online channels/social media.

## 5 Conclusions

In this multinational semi-structured interview-based study we demonstrated that C3G and primary IC-MPGN pose a substantial burden to patients and their informal caregivers. Patients frequently experience delays in diagnosis and a substantial symptomatic burden, with wide ranging impacts on physical and emotional health, work, social life and relationships. Furthermore, patients, HCPs and caregivers all reported that current treatments targeting symptoms were limited with patients finding they caused side effects which further compound the impact of the disease. Dialysis was a particular burden for patients as it is time consuming and costly. Caregivers took on a varied role in a patient’s life providing physical and psychological support as well as advocating for the patients impacting on caregiver’s own work and daily activities, HRQoL and emotional well-being. The results also provide valuable insight into HCPs perceptions and experience of the treatment pathway, highlighting a need for timely diagnosis, access to diagnostic tools, and novel targeted disease modifying treatments for C3G/primary IC-MPGN to reduce patient burden. To improve care, HCP education to recognize ambiguous symptoms as potential rare disease indicators, patient education surrounding the importance of renal biopsies, and updated treatment guidance to include emerging therapies are needed. Research in a larger population, more focused on caregivers may reveal new concepts and help to assess whether themes identified here are generalizable to the broader population.

## Data Availability

The data used in this study is available upon reasonable request by contacting the corresponding author at: mona.amet@omc.com.
